# Supplementary material for: A blueprint of the topology and mechanics of the human ovary for next-generation bioengineering and diagnosis
Source: Nat Commun. 2021 Sep 23;12:5603. doi: 10.1038/s41467-021-25934-4 (PMC8460685; doi:10.1038/s41467-021-25934-4)
Supplement: Supplementary file 1 — Supplementary information [file 41467_2021_25934_MOESM1_ESM.pdf]

Supplementary Information

A blueprint of the topology and mechanics of the human ovary

for next-generation bioengineering and diagnosis

Emna Ouni[1], Alexis Peaucelle[2,¥], Kalina T. Haas[2,¥], Olivier Van Kerk[1], Marie-Madeleine Dolmans[1,3], Timo Tuuri[4], Marjut Otala[4], Christiani A. Amorim[1,*]

[1]Pôle de Recherche en Gynécologie, Institut de Recherche Expérimentale et Clinique, Université Catholique de Louvain, 1200 Brussels, Belgium

[2]Institut Jean-Pierre Bourgin, INRAE, AgroParisTech, Université Paris-Saclay, 78000 Versailles, France

[3]Gynecology and Andrology Department, Cliniques Universitaires Saint-Luc, 1200 Brussels, Belgium

[4]Department of Obstetrics and Gynecology, Helsinki University Hospital, University of Helsinki, 00029 Helsinki, Finland

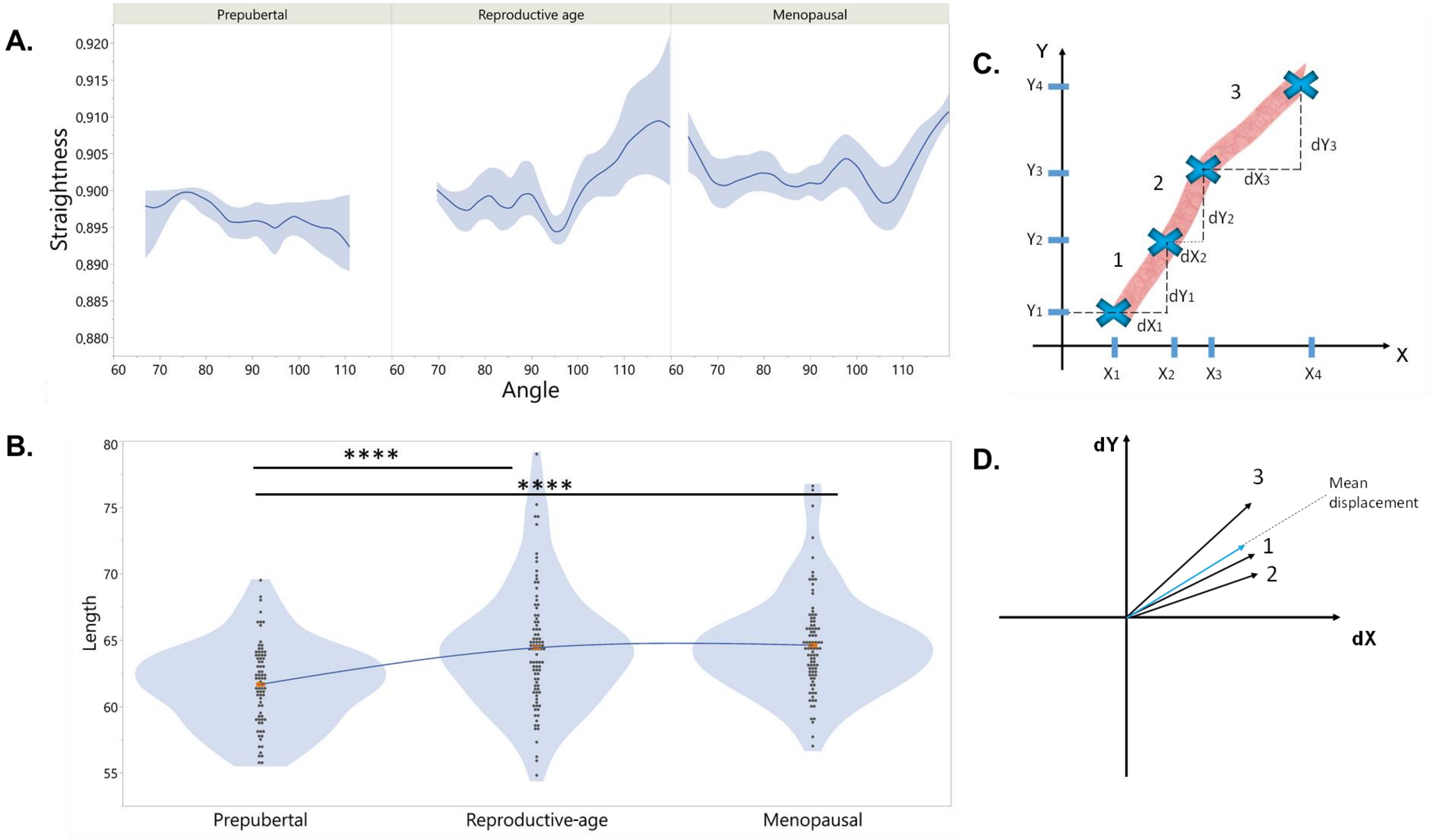
A.
Prepubertal
Reproductive age
Menopausal
Straightness
0.920
0.915
0.910
0.905
0.900
0.895
0.890
0.885
0.880
60 70 80 90 100 110
Angle
B.
Length
80
75
70
65
60
55
****
****
Prepubertal
Reproductive-age
Menopausal
C.
Y
X
Y4
Y3
Y2
Y1
X1
X2
X3
X4
1
2
3
dX1
dY1
dX2
dY2
dX3
dY3
D.
dY
dX
3
1
2
Mean
displacement

Supplementary Fig S1. Fiber straightness, length and angle measurement. Fiber straightness and length are two factors that might influence fiber tracking and orientation measurement on CT-FIRE, since long curvy fibers can lead to biased mean displacement. Based on fiber length and straightness measurement in all groups, we demonstrate that angle measurement is not influenced by these factors. A) To check whether fiber straightness impacts angle measurement, summarized statistics of straightness data were plotted against corresponding fiber orientation in each sample. No direct interdependence was noted between the two variables, which demonstrates that fiber straightness did not affect measured angles. The data plot fits a smoothing spline with a lambda value of 0.05. The curves also include the bootstrap confidence region for each fit generated by JMP Pro 14.3.0. B) Fiber length variation with age expressed in pixels. Fiber length was measured using CT-FIRE on Sirius Red-stained slides (n=5 biologically independent samples /age group). Three to five regions per slide were selected from Sirius Red scans for fiber length analysis (Full details on the number of analysed fibers are included in Table 1). The Tukey-Kramer HSD test was used to compare mean fiber lengths (orange), ****$p<0.0001$. C-D). Examples of mean displacement of a fiber trajectory and angle measurement are shown. C) Trajectory consisting of four XY coordinates; displacement from one coordinate to the next is denoted as dXY1, dXY2 and dXY3. D) The displacements (blue: dXY1, dXY2 and dXY3) are averaged to calculate the mean displacement of the trajectory (black arrow).

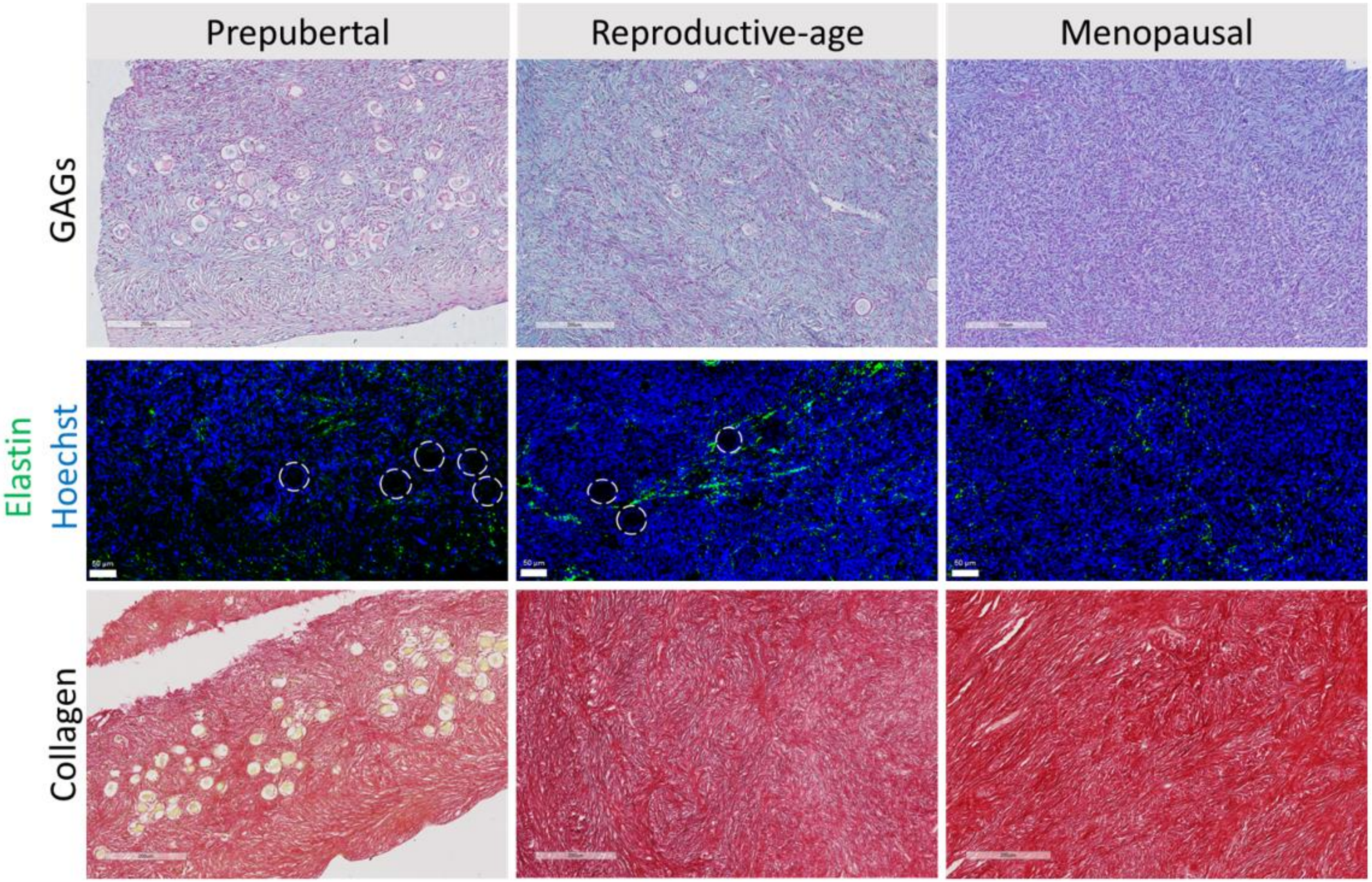

Supplementary Fig S2. Collagen, elastin and GAGs evolution with age in study subjects. Histological and elastin immunofluorescent staining of human ovarian tissue at prepubertal, reproductive and menopausal age (n=5 biologically independent samples / age group). At least 3 slides per tissue were analysed. Drawn circles highlight the presence of preantral follicles. Scale on histological staining (GAGs and Collagen): 200µm. Scale on immunofluorescent staining (elastin): 50µm.

A.

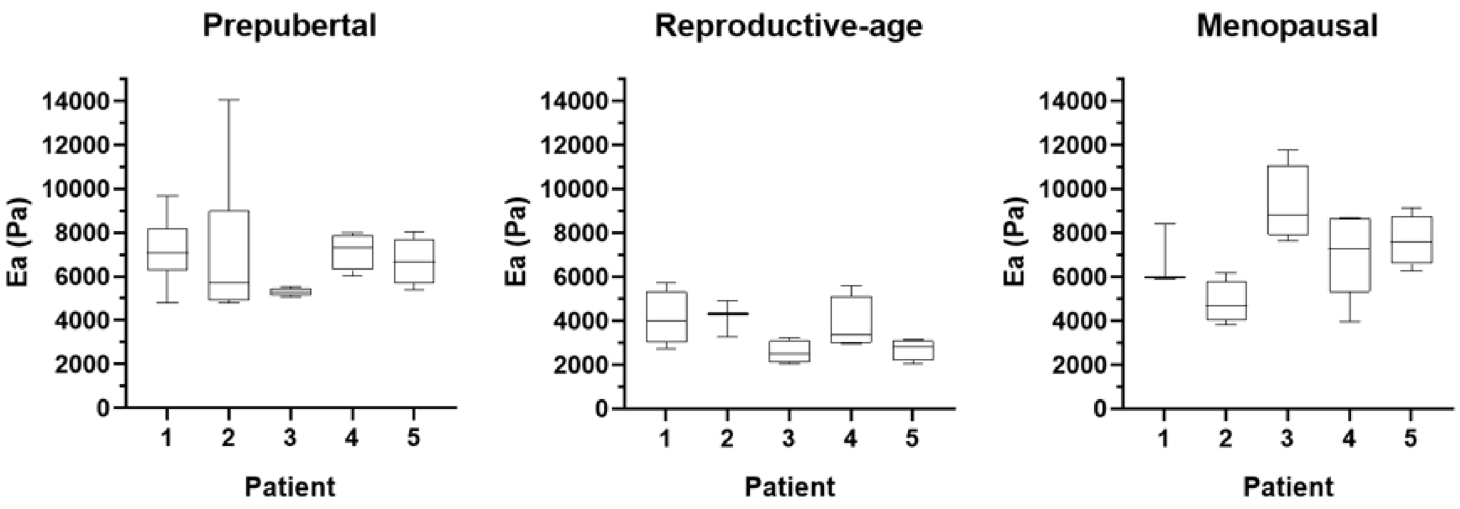
Prepubertal
Reproductive-age
Menopausal
Ea (Pa)
14000
12000
10000
8000
6000
4000
2000
0
1 2 3 4 5
Patient

B.

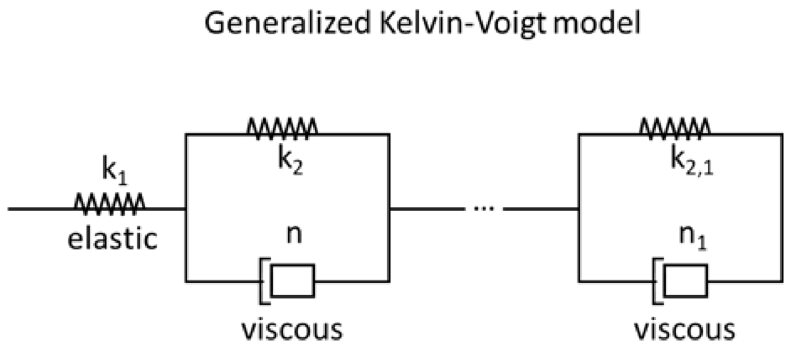
Generalized Kelvin-Voigt model
k1
elastic
k2
n
viscous
...
k2,1
n1
viscous

C.

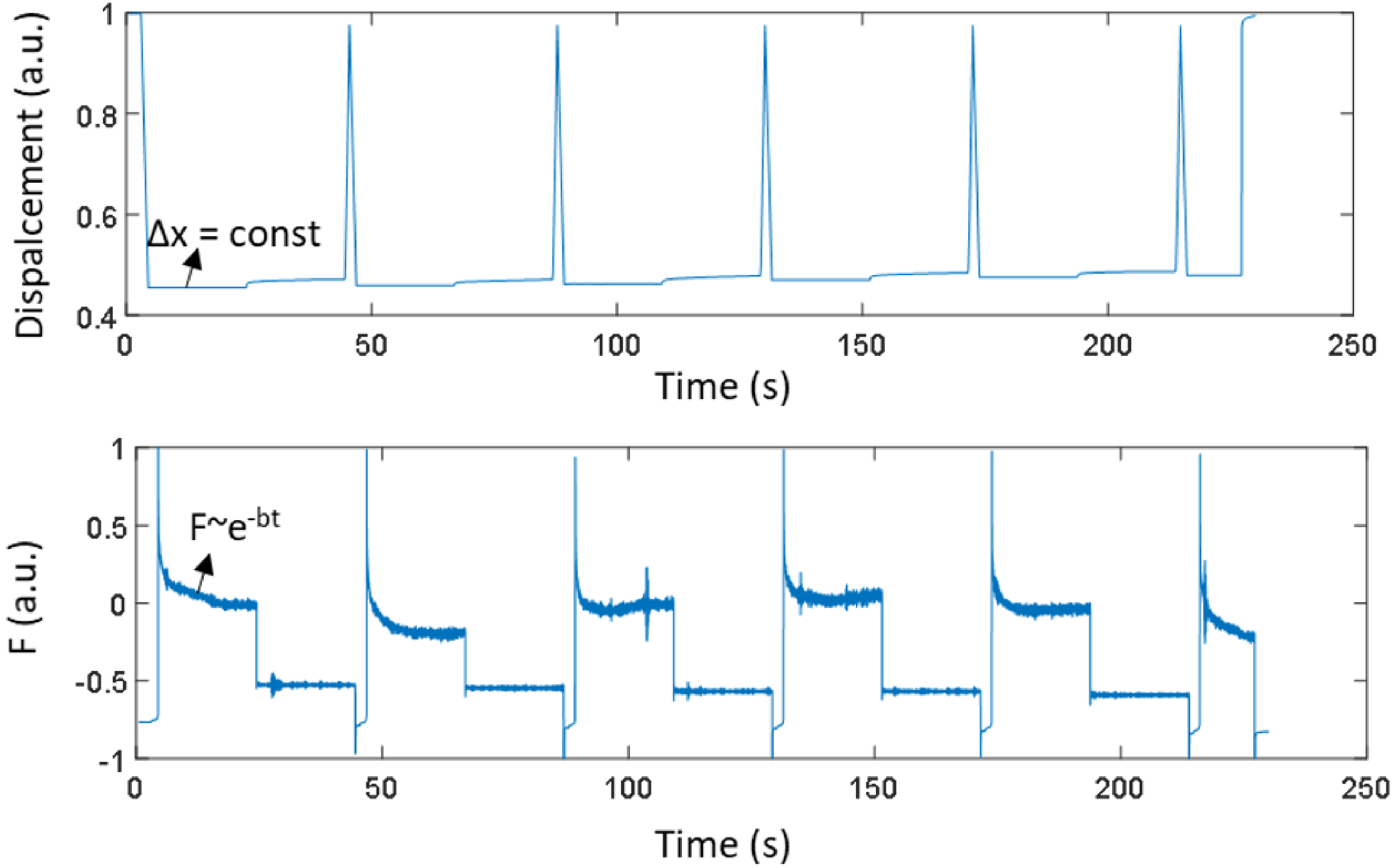

Supplementary Fig S3. Elasticity and viscoelasticity measurement details. A) Apparent elasticity (Ea) measurement in all replicates used in this study (n= 5 biologically independent samples per age group with at least 3 analyzed regions per sample). Ea was calculated over different regions in the same sample for different patients within each group. At least three maps of 100x100 µm2 area were measured per sample.

Boxplots display 25th and 75th percentile, median, and the whiskers extending to the last data point not considered outlier. B-C) Viscoelasticity measurement. Viscoelastic materials retain their shape after deformation, but with a time delay, as shown by the relaxation constant. In slow deformations, we can assume that the ECM is incompressible (Young modulus measurements). However, fast viscoelasticity measurements demonstrate that the tissue may be (reversibly) compressible in short time scales. In this context, viscoelasticity was described by the generalized Maxwell model composed of spring constants (elasticity) and dashpots (relaxation time). Relaxation time was obtained by fitting the modified Kelvin-Voigt model (spring and dashpot connected in parallel), assuming exponentially decaying force at a constant deformation [63]. Thus it permits establishing bulk elastic constant and relaxation time. The modified Kelvin-Voigt model can be described by the following:

$$F(t) = a\Delta x(1 - e^{-bt}), \text{ where } b = \frac{K_1 + k_2}{n}, \text{ and } a = \frac{K_1 k_2}{K_1 + k_2}.$$

We focused on the part of a curve where the deformation (Δx) was kept constant and the force evolved as a negative exponential. We observed that our experimental data are best fitted by two different relaxation times, which could be described by the generalized Kelvin-Voigt model. The double exponential fit modeled our data better than a single exponential [63], suggesting the presence of at least two processes behind the viscoelastic response. For more details on data processing and the model fitting, please consult the Matlab scripts at https://github.com/inatamara/AFManalysisMatlab [70].
