# Supplementary material for: A blueprint of the topology and mechanics of the human ovary for next-generation bioengineering and diagnosis
Source: Nat Commun. 2021 Sep 23;12:5603. doi: 10.1038/s41467-021-25934-4 (PMC8460685; doi:10.1038/s41467-021-25934-4)
Supplement: Supplementary file 3 — Reporting Summary [file 41467_2021_25934_MOESM3_ESM.pdf]

nature portfolio

Corresponding author(s): Christiani A. Amorim

Last updated by author(s): Aug 11, 2021

Reporting Summary

Nature Portfolio wishes to improve the reproducibility of the work that we publish. This form provides structure for consistency and transparency in reporting. For further information on Nature Portfolio policies, see our Editorial Policies and the Editorial Policy Checklist.

Statistics

For all statistical analyses, confirm that the following items are present in the figure legend, table legend, main text, or Methods section.

n/a	Confirmed	
☐	☒	The exact sample size (*n*) for each experimental group/condition, given as a discrete number and unit of measurement
☐	☒	A statement on whether measurements were taken from distinct samples or whether the same sample was measured repeatedly
☐	☒	The statistical test(s) used AND whether they are one- or two-sided<br>*Only common tests should be described solely by name; describe more complex techniques in the Methods section.*
☐	☒	A description of all covariates tested
☐	☒	A description of any assumptions or corrections, such as tests of normality and adjustment for multiple comparisons
☐	☒	A full description of the statistical parameters including central tendency (e.g. means) or other basic estimates (e.g. regression coefficient) AND variation (e.g. standard deviation) or associated estimates of uncertainty (e.g. confidence intervals)
☐	☒	For null hypothesis testing, the test statistic (e.g. *F*, *t*, *r*) with confidence intervals, effect sizes, degrees of freedom and *P* value noted<br>*Give P values as exact values whenever suitable.*
☒	☐	For Bayesian analysis, information on the choice of priors and Markov chain Monte Carlo settings
☒	☐	For hierarchical and complex designs, identification of the appropriate level for tests and full reporting of outcomes
☐	☒	Estimates of effect sizes (e.g. Cohen's *d*, Pearson's *r*), indicating how they were calculated

Our web collection on statistics for biologists contains articles on many of the points above.

Software and code

Policy information about availability of computer code

Data collection: No software was used.

Data analysis:

-Slide scan visualization
  CaseViewer V2.4 (3DHISTECH Ltd, Budapest, Hungary)
-Fiber thickness and pore characterization
  Diameter J V1.018 Plugin of Image J V1.48 (DOI: 10.1016/j.biomaterials.2015.05.015)
-Fiber orientation and straightness analyses
  CT-FIRE V2.0 Beta (DOI: 10.1117/1.JBO.19.1.016007; https://github.com/uw-loci/curvelets)
  Curvalign V4.0 Beta (DOI: 10.1117/1.JBO.19.1.016007; https://github.com/uw-loci/curvelets)
-Biomechanical analysis
  Custom-made algorithm (https://github.com/inatamara/AFManalysisMatlab)
-Topography analysis
  Mountains SEM V8.0 (commercial software)
- Statistics
  GraphPad Prism V8.0.1
  Matlab CircHist toolbox (https://github.com/zifredder/CircHist)
  Matlab circular statistics toolbox (https://github.com/circstat/circstat-matlab)
  JMP Pro V14.3.0 (in Supplementary Data)

For manuscripts utilizing custom algorithms or software that are central to the research but not yet described in published literature, software must be made available to editors and reviewers. We strongly encourage code deposition in a community repository (e.g. GitHub). See the Nature Portfolio guidelines for submitting code & software for further information.

Data

Policy information about availability of data

All manuscripts must include a data availability statement. This statement should provide the following information, where applicable:

- Accession codes, unique identifiers, or web links for publicly available datasets
- A description of any restrictions on data availability
- For clinical datasets or third party data, please ensure that the statement adheres to our policy

All analytical data associated with this study are available in the main text or supplementary materials.

Field-specific reporting

Please select the one below that is the best fit for your research. If you are not sure, read the appropriate sections before making your selection.

☒ Life sciences ☐ Behavioural & social sciences ☐ Ecological, evolutionary & environmental sciences

For a reference copy of the document with all sections, see nature.com/documents/nr-reporting-summary-flat.pdf

Life sciences study design

All studies must disclose on these points even when the disclosure is negative.

Sample size	No statistical methods were used to predetermine sample size. The scarcity of healthy human ovarian biopsies limits the number of samples available for analysis. Nevertheless, most of the differences that we observed were statistically significant, demonstrating the suitability of the sample size.
Data exclusions	In atomic force microscopy (AFM) analysis, F-z curves with no obvious contact point, clear artifacts or contact with intrafollicular areas were discarded to enable reliable and unbiased mechanical measurement of interstitial extracellular matrix.
Replication	The results reported in this manuscript were reproducible in all experiments when conducted at different time points and by various operators.<br><br>Tissues provided from the same patients (n=5 per age group) were investigated by SEM (fiber, pore and topography analyses) and AFM. A larger number of paraffin-fixed biopsies (prepubertal, n=16, reproductive-age, n=21, and menopausal, n=24) obtained from the biobank of St-Luc's Hospital were used to complete our fiber orientation evaluation.<br><br>At least three regions were acquired from each sample under SEM at 3 different magnifications: 5,000X, 12,000X and 20,000X, with reproducible results.<br><br>At least three 100 x 100 μm2 force maps and nine positions each with six repetitions of viscoelasticity measurements were reproducibly acquired on AFM.<br><br>Three to five regions per paraffine section were selected from Sirius Red scans for fiber orientation analysis.
Randomization	Patients were assigned to prepubertal, reproductive-age or menopausal groups based on their age and ovarian activity (eg. hormonal level, menorrhea).
Blinding	Analyzed samples were anonymized and a code was attributed to each sample. Thus all acquisitions were conducted without knowing the age or any clinical data to ensure objectivity of operators. Only after conducting all experiments and during statistical analyses, each sample was attributed to an age group to enable comparisons.

Reporting for specific materials, systems and methods

We require information from authors about some types of materials, experimental systems and methods used in many studies. Here, indicate whether each material, system or method listed is relevant to your study. If you are not sure if a list item applies to your research, read the appropriate section before selecting a response.

Materials & experimental systems

n/a	Involved in the study	
☐	☒	Antibodies
☒	☐	Eukaryotic cell lines
☒	☐	Palaeontology and archaeology
☒	☐	Animals and other organisms
☐	☒	Human research participants
☒	☐	Clinical data
☒	☐	Dual use research of concern

Methods

n/a	Involved in the study	
☒	☐	ChIP-seq
☒	☐	Flow cytometry
☒	☐	MRI-based neuroimaging

Antibodies

Antibodies used	In Supplementary Fig S3, the following antibodies were used:<br>Elastin (final concentration 2.2μg/mL, dilution 1:250,EPR 20603, abcam)
Validation	A negative control was conducted by adding rabbit IgG (Dako, Carpinteria, USA) instead of the primary antibody, while human testis was used for positive control.<br><br>https://www.abcam.com/elastin-antibody-epr20603-bsa-and-azide-free-ab238981.html

Human research participants

Policy information about studies involving human research participants

Population characteristics	Healthy ovarian biopsies were taken from prepubertal (mean age [±SD]=7±3 years), reproductive age (mean age [±SD]=27±5) and menopausal (mean age [±SD]=61±6 years).
Recruitment	All participating adult subjects were undergoing laparoscopic surgery for benign gynecological diseases not affecting the ovaries. Prepubertal tissue was derived from young cancer patients scheduled for ovarian cortex cryopreservation as a fertility preservation strategy, before being subjected to acute gonadotoxic cancer treatments. All samples were retrieved after obtaining patient's informed consent.<br>Patients enrolled in this study were carefully selected to avoid any possible bias or heterogeneity related to non-synchronous cycle phases between reproductive-age patients or use of hormone replacement therapy after menopause. Therefore, only fertile patients under ovarian contraceptive treatment and menopausal patients not taking hormonal replacement therapy were included in this study.
Ethics oversight	Use of human ovarian cortex was approved by the Institutional Review Board of the Université Catholique de Louvain on May 13, 2019 (IRB reference 2012/23MAR/125, registration number B403201213872).<br>Ovarian tissue from prepubertal girls was donated by patients treated at the Children's Hospital and the Department of Obstetrics and Gynecology of the University Central Hospital of Helsinki (Finland). Pediatric patients from Children's Hospital in Helsinki participated in a fertility preservation program and a research project approved by the Ethics Committee of Helsinki University Central Hospital (license number 340/13/03/03/2015). Written informed consents for pediatric patients were given by their guardians and by all age-appropriate patients.

Note that full information on the approval of the study protocol must also be provided in the manuscript.
